# Postoperative complications in patients with Behçet’s disease

**DOI:** 10.1007/s10067-024-07212-y

**Published:** 2024-10-29

**Authors:** You Jin Jung, Eun Hye Park, Ju Yeon Kim, Eun Kyoung Lee, Yunhee Choi, Eun Bong Lee

**Affiliations:** 1 Division of Rheumatology, Department of Internal Medicine, Seoul Metropolitan City Seoul Medical Center, Seoul, Republic of Korea; 2 Division of Rheumatology, Department of Internal Medicine, Chung-Ang University College of Medicine, Seoul, Republic of Korea; 3 Division of Rheumatology, Department of Internal Medicine, Chung-Ang University Gwangmyeong Hospital, Gwangmyeong, Republic of Korea; 4 Division of Rheumatology, Department of Internal Medicine, Seoul National University Hospital, Seoul, Republic of Korea; 5 Department of Molecular Medicine and Biopharmaceutical Sciences, Graduate School of Convergence Science and Technology, Seoul National University, Seoul, Republic of Korea; 6 Department of Ophthalmology, Seoul National University Hospital, Seoul, Republic of Korea; 7 Medical Research Collaborating Center, Seoul National University Hospital, Seoul, Republic of Korea; 8 Division of Rheumatology, Department of Internal Medicine, Seoul National University College of Medicine, Seoul, 03080 Republic of Korea

**Keywords:** Behçet’s disease, Cardiovascular surgery, Ophthalmic surgery, Postoperative complication, Reoperation

## Abstract

**Objective:**

To assess the proportion of postoperative complications of various surgeries in patients with Behçet’s disease (BD) and compare the risk of surgical complications between BD patients and controls.

**Methods:**

We analyzed 389 BD patients who underwent surgeries at Seoul National University Hospital between January 2003 and December 2019. Controls were 1:1 matched with BD patients based on age at surgery, sex, and type of surgery. Generalized estimating equation analyses were performed to compare the postoperative complications between BD patients and controls.

**Results:**

Among 632 surgeries in BD patients, 36 (5.7%) surgical site complications, including wound dehiscence (38.9%), bleeding (13.9%), infections (8.3%), anastomotic dehiscence (22.2%), and stricture (5.6%), occurred after median 12 days (IQR 7.8–22.0). Surgical site complications developed frequently after cardiac (33.3%) and vascular (22.2%) surgeries and rarely occurred after ophthalmic (1.5%) surgeries. Seventeen (2.7%) cases required reoperation within median 15 days (IQR 7.0–43.0). Four patients died within median 21 days (IQR 8.3–41.3 days). After adjustment for confounders, BD patients exhibited a significantly higher risk of surgical site complications (OR 3.4, 95% CI 1.4–8.0) and reoperation (OR 5.2, 95% CI 1.2–22.8) after cardiovascular surgery than controls. However, the risks of surgical site complications and reoperation after other types of surgery were similar in both groups.

**Conclusions:**

The risk of postoperative complications varies according to the type of surgeries among BD patients. While cardiovascular surgeries result in higher perioperative complications in BD patients, ophthalmic surgeries show a safety profile similar to those in non-BD patients.

**Table:** 

**Key Points** *• Patients with Behçet’s disease exhibited an increased risk of surgical site complications and subsequent reoperations after cardiovascular surgery than controls.* *• The safety profile of ophthalmic surgery was comparable between patients with Behçet’s disease and controls.* *• In patients with Behçet’s disease, the risk of postoperative complications varies according to the type of surgery.*

**Supplementary Information:**

The online version contains supplementary material available at 10.1007/s10067-024-07212-y.

## Introduction

Behçet’s disease (BD) is a chronic inflammatory disease characterized by recurrent oral and genital ulcers and ocular, musculoskeletal, cardiovascular (CV), neurological, and gastrointestinal (GI) involvement [1]. Although mucocutaneous lesions are the most common clinical features of BD, ocular and vascular involvement often results in substantial morbidity and mortality [2]. Since BD demonstrates clinical heterogeneity and unpredictable course, its treatment varies depending on the age and sex of the patient, the severity of symptoms, and organ involvement [3]. Although medical treatment is the mainstay of BD treatment, surgical intervention is also indicated in some cases with cardiac, vascular, and GI involvement, especially in cases with enlarging or ruptured aneurysms or organ-threatening ischemia [4, 5].

The outcomes of operative interventions in patients with BD exhibiting large-vessel arterial involvement vary [5–9]. In a retrospective study evaluating 47 surgical procedures for arterial aneurysms and thrombosis in 23 patients with BD during a mean follow-up of 8.4 years, the recurrence rate after the surgery was 51% [10]. While the recurrence rate in patients who received postoperative immunomodulatory treatment was 28%, the recurrence rate in untreated patients was 75% [10]. Another retrospective review of 51 surgeries for arterial aneurysms in 36 patients with BD indicated that aneurysms and thrombosis recurred after primary surgery (recurrence rates of 22% and 10%, respectively) [9]. One study indicated that patients with BD experience poor outcomes after urgent surgical interventions for pulmonary artery aneurysms, all of which resulted in death [5]. In this study, reoperations were required in three (30%) out of ten cases of primary operations for non-pulmonary arterial lesions.

Regarding surgical outcomes for GI involvement in BD, a retrospective study that assessed 41 patients with repeated bowel resections for intestinal BD indicated that patients who underwent initial surgery as an emergency procedure and those with a high perioperative erythrocyte sedimentation rate (ESR) were more likely to undergo reoperation within 6 months [11]. Another retrospective study that assessed 90 patients with intestinal BD revealed that high C-reactive protein (CRP) levels immediately after surgery were significantly associated with postoperative complications [12].

While some retrospective studies have investigated surgical outcomes in patients with BD, they lacked a control group and primarily focused on major operative interventions, such as CV or intestinal surgeries, and patients with BD experiencing severe disease, such as CV or intestinal BD [5, 8–14]. Therefore, in this study, we comprehensively evaluated the proportion of postoperative complications after various types of surgery in patients with BD experiencing different disease activities and organ involvement in a real-life clinical setting. Further, we compared the risk of postoperative complications of various surgeries between patients with BD and non-BD control populations.

## Methods

### Study population and data collection

We initially searched for patients who had an ICD 10 code for BD (M35.2x) and underwent surgeries at Seoul National University Hospital between January 2003 and December 2019. A total of 389 patients who had fulfilled the International Study Group (ISG) criteria for the diagnosis of BD [15], the International Criteria for BD (ICBD) [16], or the complete or incomplete form of the Japanese criteria [17] were included. As for controls, subjects who did not have any systemic rheumatic diseases, such as BD, rheumatoid arthritis, axial spondyloarthritis, systemic lupus erythematosus, Sjogren’s syndrome, systemic sclerosis, and systemic vasculitis, were selected and were 1:1 matched with BD patients based on age at operation (± 5 years), sex, and the type of surgery. Both patients with BD and controls had comorbidities, including hypertension (HTN), diabetes mellitus (DM), CV disease, chronic kidney disease (CKD), or malignancy.

Data on patient demographics, clinical characteristics, and comorbidities were also collected. Laboratory tests, including ESR and CRP, were performed within 3 months prior to surgery, and the results of HLA-B51 and the pathergy test at BD diagnosis were also obtained. To assess whether systemic corticosteroid (CS) use affects surgical outcomes and wound healing, we identified the prescription records of systemic CS. Since CS administration for less than 10 days reportedly does not affect wound healing in the perioperative period [18], we focused on subjects who had been prescribed CS for more than two consecutive weeks, including the day of surgery. Then, they were categorized into three groups based on daily doses of CS used: (1) CS doses of < 7.5 mg, (2) ≥ 7.5 and < 20 mg, and (3) ≥ 20 mg prednisolone equivalent. We reviewed medical records during the hospitalization period for surgery and during outpatient visits for at least 6 months after surgery. Postoperative complications included surgical site complications, subsequent reoperations owing to surgical site problems, and operation-related deaths.

### Definition of the surgical site complications

Surgical site complications were defined as composites of surgical wound and anastomotic complications. Surgical wound complications include the formation of seromas or hematomas in surgical wounds, surgical site infections, and surgical wound dehiscence, which refers to partial or total separation of the surgical wound [19]. Anastomotic complications include dehiscence of the anastomosis site and anastomotic stricture after surgery [19]. For ophthalmic surgery, we investigated the episodes of hyphema, intravitreal hemorrhage, surgical wound dehiscence, retinal detachment, and endophthalmitis.

### Statistical analysis

Data are expressed as mean ± standard deviation (SD) or median (interquartile range (IQR)) for continuous variables and number (%) for categorical variables. Since patients with BD and controls were matched and there were subjects who had undergone operations more than once, generalized estimating equation models accounting for the correlation were applied to compare the characteristics and postoperative complications between patients with BD and controls. The McNemar test was used for surgeries in which surgical site complications or reoperation did not occur. To assess whether the association between BD and postoperative complications differs depending on the type of surgery, we also tested for the interaction between surgical types (CV, abdominal, or other) and groups (patients with BD or controls). All analyses were performed using the R software (version 4.2.0) and SAS software (version 9.2; SAS Institute). Statistical significance was defined as *p* < 0.05.

## Results

### Baseline characteristics of the study population

We included 389 patients with BD in this study. The demographic and clinical characteristics of the study participants are summarized in Table 1. The mean ± SD age at diagnosis of BD was 42.9 ± 12.1 years and 44.5% were male (Table 1). A total of 235 (60.4%) patients fulfilled the ISG criteria for BD [15] and 345 (88.7%) met the ICBD [16]. Ten (2.6%) patients who did not meet both the ISG criteria and the ICBD met the incomplete form of the Japanese criteria [17]. The mean (SD) disease duration of BD was 7.0 ± 7.0 years. Most (97.2%) of the patients with BD had oral ulceration; other classical manifestations of BD included genital ulceration (55.8%), uveitis (46.0%), cutaneous lesions such as pseudofolliculitis (39.3%) and erythema nodosum (33.2%), and arthritis (23.1%) (Table 1). In addition, cardiac, vascular, GI, and neurological manifestations of BD were also shown in 4.6%, 11.8%, 19.8%, and 4.4% of the BD patients, respectively. Among 130 patients who had underwent the pathergy test, 42 (32.3%) showed positive results, and 22 out of 71 patients (31.0%) demonstrated HLA-B51 positivity (Table 1).

**Table 1:** Clinical characteristics of patients with Behçet’s disease included in the study

Characteristics	*n* = 389
Age at diagnosis, years	42.9 ± 12.1
Male sex	173 (44.5)
Disease duration, years	7.0 ± 7.0
Diagnostic criteria of BD	
International Study Group Criteria	235 (60.4)
International Criteria of Behcet’s Disease	345 (88.7)
Japanese criteria	294 (75.6)
Clinical manifestations	
Oral ulcer	378 (97.2)
Genital ulcer	217 (55.8)
Erythema nodosum	129 (33.2)
Pseudofolliculitis	153 (39.3)
Uveitis	179 (46.0)
Arthritis	90 (23.1)
Cardiac manifestations	18 (4.6)
Vascular manifestations	46 (11.8)
Gastrointestinal manifestations	77 (19.8)
Neurological manifestations	17 (4.4)
Pathergy test positivity	42/130 (32.3)
HLA-B51 positivity	22/71 (31.0)

Values are presented as mean ± standard deviation or number (%)

A total of 632 surgeries were performed in 389 patients with BD. Ophthalmic surgery (*n* = 276) was the most common surgical procedure performed in the study population. Patients with BD also received abdominal (*n* = 64), orthopedic (*n* = 55), obstetrics/gynecology (*n* = 42), vascular (*n* = 36), cardiac (*n* = 30), skin/soft tissue (*n* = 26), urologic (*n* = 25), ear, nose, and throat (ENT) (*n* = 25), thyroid (*n* = 15), neurologic (*n* = 14), general thoracic (*n* = 13), and breast (*n* = 11) surgeries (Supplementary Table S1).

### Comparison of clinical characteristics between BD patients and controls at operation

A total of 632 surgeries performed in patients with BD were 1:1 matched with those performed in controls, based on age, sex, and surgical type. The mean ± SD age at operation was 49.3 ± 12.9 years and 49.9 ± 12.8 years, in patients with BD and controls, respectively (Table 2). BD patients had significantly lower body mass index (BMI) than controls (22.6 ± 3.2 vs. 24.3 ± 4.5, *p* < 0.001). The prevalence rates of HTN (15.7% vs. 29.3%, *p* < 0.001) and DM (7.6% vs. 19.0%, *p* < 0.001) were also lower in BD patients than in controls (Table 2). A total of 303 (47.9%) patients with BD and 28 (4.4%) controls had received CS for more than two consecutive weeks, including the day of operation. Among patients with BD, CS was used for uveitis (58.1%), mucocutaneous lesions (20.8%), intestinal BD (8.6%), vascular BD (6.3%), and cardiac (5.9%), and neurological involvement (0.3%) of BD. The indications for perioperative CS use according to the CS dosage among patients with BD are presented in Supplementary Table S2. The reason for perioperative CS use among controls was as follows: The controls were prescribed CS during the perioperative period for adrenal insufficiency (*n* = 12), acute exacerbation of asthma or chronic obstructive pulmonary disease (*n* = 6), organ transplantation (*n* = 6), hematologic malignancy (*n* = 2), sudden sensorineural hearing loss (*n* = 1), and mucosal edema after turbinoplasty (*n* = 1). A total of 196 (31.0%) BD patients had used immunosuppressive agents, including azathioprine (48.5%), cyclosporine (36.2%), cyclophosphamide (6.6%), tumor necrosis factor–alpha inhibitors (6.1%), and tacrolimus (2.0%), for the treatment of following conditions: uveitis, mucocutaneous lesions, intestinal BD, vascular BD, cardiac and neurological manifestations of BD, hematologic malignancy, and organ transplantation. Six (0.9%) controls had received immunosuppressants due to organ transplantation. As expected, BD patients demonstrated higher levels of ESR (22.3 ± 21.2 vs. 18.9 ± 18.8 mm/h, *p* = 0.008) and serum CRP levels (1.2 ± 3.6 vs. 0.5 ± 1.7 mg/dL, *p* < 0.001) before the surgery than controls (Table 2). The proportion of participants with high inflammatory markers was also higher among BD patients than controls (58.1% vs. 46.3%, *p* < 0.001).

**Table 2:** Comparison of clinical characteristics between patients with BD and controls at surgery

Characteristics	BD patients (*n* = 632)^a^	Controls (*n* = 632)^b^	*p* value^c^
Age at surgery, years	49.3 ± 12.9	49.9 ± 12.8	
Male sex	295 (46.7)	295 (46.7)	
Body mass index, kg/m^2^	22.6 ± 3.2	24.3 ± 4.5	< 0.001
Comorbidities			
Hypertension	99 (15.7)	185 (29.3)	< 0.001
Diabetes mellitus	48 (7.6)	120 (19.0)	< 0.001
Cardiovascular disease	86 (13.6)	70 (11.1)	0.109
Chronic kidney disease^d^	13 (2.1)	29 (4.6)	0.126
Malignancy	53 (8.4)	61 (9.7)	0.485
Perioperative corticosteroid use^e^	303 (47.9)	28 (4.4)	< 0.001
Dosage of prednisolone equivalent^e^			0.378
< 7.5 mg/day	84 (27.7)	11 (39.3)	
≥ 7.5 and < 20 mg/day	97 (32.0)	7 (25.0)	
≥ 20 mg/day	122 (40.3)	10 (35.7)	
Perioperative Immunosuppressant use	196 (31.0)	6 (0.9)	< 0.001
Cyclophosphamide	13 (6.6)	0 (0)	
Azathioprine	95 (48.5)	2 (33.3)	
Cyclosporine	72 (36.2)	4 (66.7)	
Tacrolimus	4 (2.0)	0 (0)	
Tumor necrosis factor–alpha inhibitor	12 (6.1)	0 (0)	
Laboratory findings before surgery^f^			
Erythrocyte sedimentation rate, mm/h	22.3 ± 21.2	18.9 ± 18.8	0.018
C-reactive protein, mg/dL	1.2 ± 3.6	0.5 ± 1.7	< 0.001

Values are presented as mean ± standard deviation or number (%)

^a^632 surgeries in 389 patients with BD

^b^632 surgeries in 628 controls

^c^Generalized estimating equation with a sandwich estimator to account for correlation within pairs or subjects

^d^Glomerular filtration rate < 45 mL/min

^e^Corticosteroid use during 2 consecutive weeks, including the day of surgery

^f^Performed within 3 months prior to surgery

### Postoperative surgical site complications

Among 632 surgeries, 36 (5.7%) surgical site complications had occurred after median 12 days (IQR 7.8–22.0 days) from operation among BD patients; 30 (4.7%) surgical site complications had developed within median 12 days (IQR 6–22 days) among controls. The surgical site complications in patients with BD included wound dehiscence (38.9%), bleeding (13.9%), surgical site infections (8.3%), anastomotic dehiscence (22.2%), and stricture (5.6%) (Supplementary Table S3). The proportions of surgical site complications according to the type of surgery are summarized in Table 3 and Supplementary Table S1. When analyzed by the type of surgeries, surgical site complications developed most frequently after the cardiac (33.3%) surgery, followed by vascular (22.2%), abdominal (12.5%), ENT (8.0%), skin/soft tissue (7.7%), urological (4.0%), obstetrics/gynecological (2.4%), and ophthalmic (1.5%) surgeries among patients with BD (Table 3). Among 30 cardiac surgeries, 10 (33.3%) surgical site complications were identified in patients with BD, while only 3 (10.0%) were found in controls (Table 3). Consequently, the risk of surgical site complications after the cardiac surgery was 4.5 times higher among BD patients compared with controls (95% CI 1.5–13.6; *p* = 0.008) (Table 3). Even after adjusting for confounders, including BMI, comorbidities of HTN, DM, CV disease, CKD, and malignancy, and CS dose, BD patients demonstrated a significantly higher risk of surgical site complications after CV surgery (OR 3.4 (95% CI 1.4–8.0), *p* = 0.005) compared with controls (Table 4).

**Table 3:** Postoperative surgical site complications, subsequent reoperation, and operation-related death

Type of surgery	Surgical site complications				Reoperation				Operation-related death		
	Proportion		OR (95% CI)^a^	*p* value	Proportion		OR (95% CI)^a^	*p* value	Proportion		*p* value
	Patients	Controls			Patients	Controls			Patients	Controls	
Cardiac (30)	10 (33.3)	3 (10.0)	4.5 (1.5–13.6)	0.008	5 (16.7)	1 (3.3)	5.4 (0.7–41.4)	0.105	3 (10.0)	0 (0.0)	0.083^b^
Vascular (36)	8 (22.2)	4 (11.1)	2.3 (0.7–7.8)	0.199	6 (16.7)	2 (5.6)	3.1 (0.7–14.3)	0.140	0 (0.0)	0 (0.0)	
Abdominal (64)	8 (12.5)	5 (7.8)	1.7 (0.6–4.6)	0.314	5 (7.8)	2 (3.1)	2.6 (0.5–15.0)	0.273	0 (0.0)	0 (0.0)	
Ophthalmic (276)	4 (1.5)	6 (2.2)	0.7 (0.2–2.1)	0.478	1 (0.4)	5 (1.8)	0.2 (0.0–1.0)	0.051	0 (0.0)	0 (0.0)	
Ear, nose, and throat (25)	2 (8.0)	1 (4.0)	2.0 (0.2–22.4)	0.563	0 (0.0)	1(4.0)	Not estimable	0.317^b^	0 (0.0)	0 (0.0)	
Skin and soft tissue (26)	2 (7.7)	4 (15.4)	0.5 (0.1–2.1)	0.315	0 (0.0)	0 (0.0)	Not estimable		0 (0.0)	0 (0.0)	
Neurologic (14)	0 (0.0)	1 (7.1)	Not estimable	0.317^b^	0 (0.0)	1 (7.1)	Not estimable	0.317^b^	0 (0.0)	0 (0.0)	
Urologic (25)	1 (4.0)	0 (0.0)	Not estimable	0.317^b^	0 (0.0)	0 (0.0)	Not estimable		0 (0.0)	0 (0.0)	
Obstetrics/gynecology (42)	1 (2.4)	3 (7.3)	0.3 (0.0–3.3)	0.329	0 (0.0)	0 (0.0)	Not estimable		0 (0.0)	0 (0.0)	
Orthopedic (55)	0 (0.0)	0 (0.0)	Not estimable		0 (0.0)	0 (0.0)	Not estimable		0 (0.0)	0 (0.0)	
Thoracic (13)	0 (0.0)	0 (0.0)	Not estimable		0 (0.0)	0 (0.0)	Not estimable		1 (7.7)	0 (0.0)	0.317^b^
Thyroid (15)	0 (0.0)	0 (0.0)	Not estimable		0 (0.0)	0 (0.0)	Not estimable		0 (0.0)	0 (0.0)	
Breast (11)	0 (0.0)	3 (27.3)	Not estimable	0.083^b^	0 (0.0)	1 (9.1)	Not estimable	0.317^b^	0 (0.0)	0 (0.0)	

*OR* odds ratio, *CI* confidence interval

^a^Generalized estimating equation with a sandwich estimator to account for correlation within pairs or subjects

^b^McNemar test was used

**Table 4:** Multivariable analysis for postoperative surgical site complications and subsequent reoperations

Type of surgery	Surgical site complications					Reoperations				
	Adjusted proportion (95% CI)^a^		Adjusted OR (95% CI)^a,^^b^	*p* value	*p* value^c^	Adjusted proportion (95% CI)^a^		Adjusted_OR (95% CI)^a,^^b^	*p* value	*p* value^c^
	Patients	Controls				Patients	Controls			
Cardiovascular	22.7 (12.0–39.0)	8.0 (3.2–18.8)	3.4 (1.4–8.0)	0.005	0.005	23.2 (11.8–40.6)	5.5 (1.3–20.2)	5.2 (1.2–22.8)	0.028	0.006
Abdominal	12.8 (6.1–24.8)	7.6 (3.2–16.9)	1.8 (0.6–5.1)	0.275		8.6 (3.4–19.8)	3.3 (0.9–11.7)	2.8 (0.5–14.9)	0.229	
Others	1.9 (1.0–3.7)	3.8 (2.4–5.9)	0.5 (0.2–1.1)	0.083		0.2 (0.0–1.3)	1.4 (0.7–3.0)	0.1 (0.0–0.9)	0.040	

*OR* odds ratio, *CI* confidence interval

^a^Adjusted for body mass index, comorbidities (hypertension, diabetes mellitus, cardiovascular disease, chronic kidney disease, and malignancy), and corticosteroid dose

^b^Generalized estimating equation with a sandwich estimator to account for correlation within pairs or subjects

^c^*p* value for the interaction between surgical type and group (BD patients or controls)

Regarding other types of surgeries, there was no significant difference between patients with BD and controls in terms of the risk of surgical site complications in both analyses, with and without adjustment for confounders (Table 3). Ophthalmic surgeries resulted in four (1.5%) postoperative surgical site complications (Table 3), including two cases of retinal detachment and one case of hyphema and intravitreal hemorrhage each (Supplementary Table S3). All four cases of postoperative complications were associated with underlying Behçet’s uveitis. The association between BD and surgical site complications differed significantly depending on the type of surgery (CV, abdominal, or other) (*p* = 0. 0.005) (Table 4).

### Subsequent reoperation

Among 632 surgeries, 17 (2.7%) cases required reoperation due to surgical site problems within median 15 days (IQR 7.0–43.0 days) among patients with BD; 13 (2.1%) resulted in subsequent reoperation within median 19 days (IQR 8–22 days) among controls. Reoperation was performed after the cardiac (16.7%), vascular (16.7%), abdominal (7.8%), or ophthalmic (0.4%) surgeries in BD patients (Table 3). After adjusting for confounders, BD patients showed a significantly higher risk of reoperation after CV surgery (OR 5.2, 95% CI 1.2–22.8; *p* = 0.028) than controls (Table 4). The association between BD and reoperation after initial surgery differed significantly depending on the surgical type (*p* = 0.006) (Table 4). No significant association was found between BD and reoperation after other types of surgery.

### Operation-related death

A total of four deaths (0.6%) in association with surgery were identified among BD patients within median 21 days (IQR 8.3–41.3 days) whereas no operation-related death occurred among controls. In our study population, cardiac surgery was the leading cause of death (75%), and general thoracic surgery also resulted in operation-related death in one patient (Table 3).

### Risk factors for surgical site complications of CV surgery in BD patients

To identify factors associated with surgical site complications after CV surgery among BD patients, we performed a subgroup analysis of BD patients with CV surgeries. High CRP levels prior to surgery (OR 3.2 (95% CI 1.1–9.6), *p* = 0.036) and comorbidity of DM (OR 6.4 (95% CI 1.4–28.6), *p* = 0.015) were significantly associated with development of surgical site complications after CV surgery among BD patients (Supplementary Table S4). However, age at surgery, sex, disease duration, BMI, CS dosage, and use of immunosuppressive agents were not significantly associated with surgical site complications.

## Discussion

In this study, we evaluated the proportion of postoperative complications in various surgeries in patients with BD and compared the risk of surgical complications between patients with BD and controls. While patients with BD showed a significantly higher risk of both surgical site complications and subsequent reoperation after CV surgery than controls, there was no difference in the risk of postoperative complications after other types of surgeries between patients with BD and controls. Surgery-related death occurred in four (0.6%) cases among BD patients within median 21 days, whereas no deaths were observed among controls.

A previous systematic review and meta-analysis of BD and metabolic syndrome revealed that patients with BD had an increased risk of HTN and DM [20]. However, in the current study, the prevalence rates of HTN and DM were lower in patients with BD than in the controls. Several factors should be considered when interpreting these results. First, it is well known that a higher BMI is associated with an increased risk of HTN and DM [21]. A lower BMI among patients with BD might have led to a lower prevalence of HTN and DM in patients with BD than in controls in our study. Second, while significant correlation between HTN and vascular BD was described previously [22], the proportion of BD patients with vascular manifestations was only 11.8% in this study. Third, higher lipid profiles and BMI were observed in patients with BD with a longer disease duration (> 10 years) than in those with a shorter BD duration (< 10 years) [23]. Given that the mean duration of BD in our study population was 7 years, we presume that the prevalence of HTN and DM might increase as the disease duration increases.

The pathergy phenomenon, which refers to skin hyperreactivity to trauma, is one of the unique clinical characteristics of BD and is associated with a delayed wound healing process [24, 25]. Although the exact mechanism is unclear, skin injury in patients exhibiting pathergy is thought to trigger an inflammatory response by increasing the release of inflammatory cytokines from keratinocytes and inappropriately activating mononuclear cells, resulting in perivascular infiltrations that are characteristically observed in histopathological studies [26–28]. Therefore, there have been concerns regarding postoperative complications in patients with BD undergoing surgical procedures. In a retrospective review of 43 surgeries performed in 37 patients with BD, a significantly higher rate of postoperative complications was reported in patients with a positive pathergy test than in those with a negative result [29]. In our study population, no significant association was observed between the postoperative complications and positive pathergy test results.

The postoperative course of vascular surgery in patients with BD and arterial lesions is often complicated by aneurysm recurrence and graft occlusion, resulting in a high mortality rate [30]. Regarding long-term outcomes of vascular surgery in BD patients, the 10-year survival rate was reported as 30%, the 10-year complication-free survival rate was 13%, and the 5-year reoperation-free survival rate was 26% [4]. Another retrospective study evaluating 59 CV surgeries performed in 41 BD patients revealed that postoperative complications including paravalvular leakage, anastomotic dehiscence, fistula, graft occlusion, and pseudoaneurysmal formations developed in 49.2% during mean 65.3 ± 48.1 months [31]. Previous studies have reported poor outcomes of CV surgery among BD patients [5–8, 30, 31]; however, none has compared the postoperative courses of CV surgery between patients with BD and non-BD control populations. Our study showed that BD patients had 3.4 times higher risk of surgical site complications and 5.2 times higher risk of subsequent reoperations after initial CV surgery than controls after adjustment for confounders. In addition, in line with previous studies reporting high mortality rate associated with CV surgeries among BD patients [2, 31], three (10%) cardiac surgeries resulted in operation-related deaths within median 11 days among BD, whereas no deaths were identified among controls.

Surgical treatment for intestinal BD is usually reserved for patients refractory to medical treatment or with intestinal perforation, obstruction, fistula formation, or severe GI bleeding [32]. Previously, few studies have reported poor clinical outcomes of surgical interventions among patients with intestinal BD [11, 32, 33]. Postoperative complications, including anastomotic leakage, fistula formation, wound infection, intra-abdominal abscess, bleeding, and ileus, were identified in 30.8% of intestinal BD patients with intestinal surgery, and death occurred in 3.3% related to postoperative sepsis [33]. The reoperation rate among BD patients who had undergone initial bowel surgery is reportedly high, ranging from 30 to 44%, and ulcers frequently recur at or near the anastomosis site [32]. In the current study, the risk of surgical site complications and reoperation after intestinal surgery was numerically higher among patients with BD than among controls, but the difference was not statistically significant due to the small sample size. Further studies with larger populations are required to confirm these findings.

Several studies have investigated the factors responsible for postoperative complications in patients with BD. The use of immunosuppressive agents before or after surgery significantly reduced the occurrence of postoperative complications after CV surgery among patients with BD [9, 10, 31]. High perioperative inflammatory markers, including ESR [11] and CRP [12], have been shown to be significant risk factors for postoperative complications after bowel surgery in patients with intestinal BD. Emergency surgery has also been reported as a poor prognostic factor for early reoperation within 6 months of the initial surgery among patients with intestinal BD who underwent bowel surgery [11]. We found that the association between BD and postoperative complications differed significantly depending on the type of surgery. Although CV surgeries resulted in more surgical site complications and subsequent reoperations in patients with BD than in controls, other types of surgeries were unrelated to postoperative complications in patients with BD. Moreover, high CRP levels before surgery, which may reflect the higher disease activity of BD, were significantly associated with postoperative complications after CV surgery in patients with BD. Previously, in a meta-analysis and systematic review, DM was reported as a significant risk factor for postoperative complications, including infections, wound healing disorders, hematoma, reoperation, and mortality after non-cardiac surgery [34]. Similarly, DM was identified as a significant risk factor for surgical site complications after CV surgery in patients with BD (Supplementary Table S4). However, demographic factors, BD duration, CS dosage, and immunosuppressant use were not significantly associated with surgical site complications.

Among 276 ophthalmic surgeries, three postoperative complications developed after vitrectomy in patients with Behçet’s uveitis and one after glaucoma surgery, which was complicated by Behçet’s uveitis. Behçet’s uveitis potentially has high morbidity due to its explosive and recurrent nature [35], and a few small retrospective studies have reported the surgical outcomes of vitrectomy for ocular complications of BD [36, 37]. A retrospective study including 15 patients with ocular BD showed that visual acuity increased in half of the cases and uveitis attacks significantly decreased after vitrectomy [36]. In another study involving 12 patients with ocular BD, vision was restored or stabilized in 42.9%, immunosuppressive agents reduced in 41.6%, and no serious ocular inflammatory episodes were shown during the median follow-up of 105 months after the vitrectomy [37]. Our study had a larger sample size with a control group, and ophthalmic surgeries showed relatively favorable outcomes among patients with BD.

Based on these results, we should be reassured that ophthalmic surgeries are relatively safe in BD. However, special perioperative care should be provided to patients with BD who are undergoing CV surgery. Although more studies are warranted to confirm our findings and understand the mechanisms, we assume that direct vessel wall interventions cause poorer outcomes than non-vascular surgeries among patients with BD, given that vasculitis and vascular hyperreactivity are distinct pathological characteristics of BD [38].

This study has several strengths that deserve comments. First, this is the first study to assess the comparative risk of postoperative complications after various surgical interventions in patients with BD and controls. This study included a control group that underwent both major and minor surgeries, allowing a more comprehensive review of the natural course of surgical interventions among patients with BD. Second, we included patients with BD who had diverse clinical manifestations and disease activities. Because the ISG criteria for BD do not account for CV or neurological involvement [15], and both the ISG and ICBD do not include intestinal BD [15, 16], we have also applied the Japanese criteria [17], so as not to overlook patients with CV (16.4% in our study population), intestinal (19.8%), or neurological (4.4%) involvement.

However, this study has several limitations. First, as with previous studies, this study was designed retrospectively and had a relatively small sample size owing to the low prevalence of BD. Second, we obtained laboratory results performed within 3 months before surgery, which might be less relevant than those acquired shortly before surgery, to evaluate their impact on surgical outcomes. However, in the real-world clinical setting, most patients were followed up at 3-month intervals in the outpatient clinic, and those with mild disease often do not undergo a blood test just before surgical procedures. Third, we did not investigate long-term surgical outcomes, as the time period of interest for postoperative complications was 6-month postoperative periods. However, it was previously reported that the incidence of postoperative complications was the highest within 6 months of surgery in patients with BD [29]. Further studies are required to examine long-term outcomes of various surgeries in patients with BD. Fourth, we included various classification criteria for BD to recruit patients with diverse clinical features; the study outcomes might have been different if we had only adopted ISG or ICBD alone.

## Conclusions

In summary, we found that patients with BD had an increased risk of surgical site complications and subsequent reoperations after CV surgery compared with controls. In contrast, minor surgeries, such as ophthalmic surgeries, are relatively safe for patients with BD. Further studies with larger populations are required to validate our results.

## Supplementary Information

Below is the link to the electronic supplementary material.

Supplementary file1 (DOCX 34 KB)

## Data Availability

The datasets used and analyzed in the current study are available from the corresponding author upon reasonable request.

## Abbreviations

BD: Behçet’s disease
CV: Cardiovascular
GI: Gastrointestinal
ESR: Erythrocyte sedimentation rate
CRP: C-reactive protein
ISG: International Study Group
ICBD: International Criteria for Behçet’s Disease
HTN: Hypertension
DM: Diabetes mellitus
CKD: Chronic kidney disease
CS: Corticosteroids
SD: Standard deviation
IQR: Interquartile range
ENT: Ear, nose, and throat
BMI: Body mass index
