# Supplementary material for: Postoperative complications in patients with Behçet’s disease
Source: Clin Rheumatol. 2024 Oct 29;43(12):3829–38. doi: 10.1007/s10067-024-07212-y (PMC11582265; doi:10.1007/s10067-024-07212-y)
Supplement: Supplementary file 1 — Supplementary file1 (DOCX 34 KB) [file 10067_2024_7212_MOESM1_ESM.docx]

**Supplementary Tables**

**Supplementary Table S1. The proportion of the surgical site complications, subsequent reoperations, and operation-related death by specific type of surgeries**

| **Type of surgery** | **Surgical site complications** | | | **Reoperations** | | | **Death (%)** | |
| --- | --- | --- | --- | --- | --- | --- | --- | --- |
|  | **Patient** | **Control** | **Patient** | | **Control** | **Patient** | | **Control** |
| Cardiac | 10/30 (33.3) | 3/30 (10.0) | 5/30 (16.7) | | 1/30 (3.3) | 3/30 (10.0) | | 0/30 (0.0) |
| Valvular | 10/26 (38.5) | 3/26 (11.5) | 5/26 (19.2) | | 1/26 (3.8) | 3/26 (11.5) | | 0/26 (0.0) |
| CABG | 0/3 (0.0) | 0/3 (0.0) | 0/3 (0.0) | | 0/3 (0.0) | 0/3 (0.0) | | 0/3 (0.0) |
| Congenital | 0/1 (0.0) | 0/1 (0.0) | 0/1 (0.0) | | 0/1 (0.0) | 0/1 (0.0) | | 0/1 (0.0) |
| Vascular | 8/36 (22.2) | 4/36 (11.1) | 6/36 (16.7) | | 2/36 (5.6) | 0/36 (0.0) | | 0/36 (0.0) |
| Aortic | 5/19 (26.3) | 3/19 (15.8)) | 3/19 (15.8) | | 2/19 (10.5) | 0/19 (0.0) | | 0/19 (0.0) |
| Carotid | 1/2 (50.0) | 0/2 (0.0) | 0/2 (0.0) | | 0/2 (0.0) | 0/2 (0.0) | | 0/2 (0.0) |
| Neurologic | 1/9 (11.1) | 0/9 (0.0) | 2/9 (11.1) | | 0/9 (0.0) | 0/9 (0.0) | | 0/9 (0.0) |
| Peripheral | 1/6 (16.7) | 1/6 (16.7) | 1/6 (16.7) | | 0/6 (0.0) | 0/6 (0.0) | | 0/6 (0.0) |
| Abdominal | 8/64 (12.5) | 5/64 (7.8) | 5/64 (7.8) | | 2/64 (3.1) | 0/64 (0.0) | | 0/64 (0.0) |
| Gastric | 1/3 (33.3) | 1/3 (33.3) | 0/3 (0.0) | | 0/3 (0.0) | 0/3 (0.0) | | 0/3 (0.0) |
| Intestinal | 5/39 (12.8) | 4/39 (10.3) | 3/39 (7.7) | | 2/39 (5.1) | 0/39 (0.0) | | 0/39 (0.0) |
| Hepatobiliary | 1/11 (9.1) | 0/11 (0.0) | 1/11 (9.1) | | 0/11 (0.0) | 0/11 (0.0) | | 0/11 (0.0) |
| Others | 1/11 (9.1) | 0/11 (0.0) | 1/11 (9.1) | | 0/11 (0.0) | 0/11 (0.0) | | 0/11 (0.0) |
| Ophthalmic | 4/276 (1.5) | 6/276 (2.2) | 1/276 (0.4) | | 5/276 (1.8) | 0/276 (0.0) | | 0/276 (0.0) |
| Cataract | 0/143 (0.0) | 1/143 (0.7) | 0/143 (0) | | 1/143 (0.7) | 0/143 (0.0) | | 0/143 (0.0) |
| Vitrectomy | 3/79 (3.8) | 3/79 (3.8) | 1/79 (1.3) | | 3/79 (3.8) | 0/79 (0.0) | | 0/79 (0.0) |
| Glaucoma | 1/37 (2.7) | 1/37 (2.7) | 0/37 (0.0) | | 0/37 (0.0) | 0/37 (0.0) | | 0/37 (0.0) |
| Cornea | 0/8 (0.0) | 1/8 (12.5) | 0/8 (0.0) | | 1/8 (12.5) | 0/8 (0.0) | | 0/8 (0.0) |
| Others | 0/9 (0.0) | 0/9 (0.0) | 0/9 (0.0) | | 0/9 (0.0) | 0/9 (0.0) | | 0/9 (0.0) |
| Ear, nose, and throat | 2/25 (8.0) | 1/25 (4.0) | 0/25 (0.0) | | 1/25 (4.0) | 0/25 (0.0) | | 0/25 (0.0) |
| Skin and soft tissue | 2/26 (7.7) | 4/26 (15.4) | 0/26 (0.0) | | 0/26 (0.0) | 0/26 (0.0) | | 0/26 (0.0) |
| Neurologic | 0/14 (0.0) | 1/14 (7.1) | 0/14 (0.0) | | 1/14 (7.1) | 0/14 (0.0) | | 0/14 (0.0) |
| Brain | 0/3 (0.0) | 1/3 (33.3) | 0/3 (0.0) | | 1/3 (33.3) | 0/3 (0.0) | | 0/3 (0.0) |
| Spine | 0/11 (0.0) | 0/11 (0.0) | 0/11 (0.0) | | 0/11(0) | 0/11 (0.0) | | 0/11 (0.0) |
| Urologic | 1/25 (4.0) | 0/25 (0.0) | 0/25 (0.0) | | 0/25 (0.0) | 0/25 (0.0) | | 0/25 (0.0) |
| Obstetrics/Gynecology | 1/42 (2.4) | 3/42 (7.3) | 0/42 (0.0) | | 0/42 (0.0) | 0/42 (0.0) | | 0/42 (0.0) |
| Orthopedic | 0/55 (0.0) | 0/55 (0.0) | 0/55 (0.0) | | 0/55 (0.0) | 0/55 (0.0) | | 0/55 (0.0) |
| General thoracic | 0/13 (0.0) | 0/13 (0.0) | 0/13 (0.0) | | 0/13 (0.0) | 1/13 (7.7) | | 0/13 (0.0) |
| Thyroid | 0/15 (0.0) | 0/15 (0.0) | 0/15 (0.0) | | 0/15 (0.0) | 0/15 (0.0) | | 0/15 (0.0) |
| Breast | 0/11 (0.0) | 3/11 (27.3) | 0/11 (0.0) | | 1/11 (9.1) | 0/11 (0.0) | | 0/11 (0.0) |
| **Total** | **36/632 (5.7)** | **30/632 (4.7)** | **17/632 (2.7)** | | **13/632 (2.1)** | **4/632 (0.6)** | | **0/632 (0.0)** |

Values are presented as number of events/number of surgeries (%).

**Supplementary Table S2. The indications for perioperative corticosteroid use according to the corticosteroid dosage among patients with Behcet’s disease**

| **Dosage of prednisolone equivalent^a^** | **Indications** | **n=303** |
| --- | --- | --- |
| <7.5 mg/day | Mucocutaneous lesions | 54 |
|  | Uveitis | 19 |
|  | Intestinal BD | 6 |
|  | Vacular BD | 4 |
|  | Cardic manifestations | 1 |
| ≥7.5 and <20 mg/day | Uveitis | 81 |
|  | Mucocutaneous lesions | 8 |
|  | Intestinal BD | 3 |
|  | Cardic manifestations | 2 |
|  | Neurologic manifestations | 1 |
|  | Vascular BD | 2 |
| ≥20 mg/day | Uveitis | 76 |
|  | Intestinal BD | 18 |
|  | Cardiac involvement | 14 |
|  | Vascular BD | 13 |
|  | Cutaneous lesions | 1 |

^a^Corticosteroid use during 2 consecutive weeks including the day of surgery

**Supplementary Table S3. Specific type of surgical site complications among patients with Behcet’s disease and controls**

| **Type of complications** | **BD patients**  (n=36) | **Controls**  (n=30) |
| --- | --- | --- |
| Non-ophthalmic surgery |  |  |
| Wound dehiscence | 14 | 8 |
| Bleeding | 5 | 5 |
| Seroma | 0 | 4 |
| Wound infection | 3 | 3 |
| Anastomotic dehiscence | 8 | 2 |
| Anastomotic stricture | 2 | 2 |
| Ophthalmic surgery |  |  |
| Hyphema | 1 | 3 |
| Intravitreal hemorrhage | 1 | 0 |
| Wound dehiscence | 0 | 2 |
| Retinal detachment | 2 | 1 |

Values are presented as number of events.

**Supplementary Table S4. Risk factors associated with surgical site complications after cardiovascular surgery in patients with Behcet’s disease.**

| **Variables** | **Univariable analysis** | | |
| --- | --- | --- | --- |
|  | **OR**^a^ | **(95% CI)** | ***P* value** |
| Age at operation | 0.96 | (0.91-1.01) | 0.101 |
| Disease duration | 0.97 | (0.89-1.06) | 0.456 |
| Male sex | 2.10 | (0.66-6.72) | 0.211 |
| Body mass index | 0.91 | (0.78-1.06) | 0.243 |
| Diabetes mellitus | 6.43 | (1.44-28.62) | 0.015 |
| Use of immunosuppressive agent | 1.46 | (0.43-4.93) | 0.543 |
| Dosage of prednisolone equivalent^b^ |  |  |  |
| <7.5 mg/day | 1 |  |  |
| ≥7.5 and <20 mg/day | 1.67 | (0.07-37.73) | 0.748 |
| ≥20 mg/day | 1.25 | (0.10-15.44) | 0.862 |
| CRP at operation^c^ |  |  |  |
| Normal | 1 |  |  |
| High | 3.21 | (1.08-9.55) | 0.036 |

OR, odds ratio; CI, confidence interval; ESR, erythrocyte sedimentation rate; CRP, C-reactive protein.

^a^Generalized estimating equation with a sandwich estimator to account for correlation within pairs or subjects.

^b^Corticosteroid use during 2 consecutive weeks including the day of surgery.

^c^Performed within three months prior to surgery.
